# Psychiatric Risk Governance Across Jurisdictions: A Comparative Analysis of Involuntary Treatment, Community Treatment Orders, and Forensic Mental Health Services

**DOI:** 10.3390/healthcare13182363

**Published:** 2025-09-20

**Authors:** Matteo Lippi, Laura Leondina Campanozzi, Giuseppe D’Andrea, Donato Morena, Francesca Orsini, Felice Marco Damato, Giuseppe Fanelli, Yasin Hasan Balcioglu, Howard Ryland, Thomas Fovet, Birgit Völlm, Javier Vicente-Alba, Charles L. Scott, Paola Frati, Vittoradolfo Tambone, Raffaella Rinaldi

**Affiliations:** 1 Department of Anatomical, Histological, Forensic and Orthopedic Sciences, Sapienza University of Rome, 00185 Rome, Italy; matteo.lippi@uniroma1.it (M.L.); donato.morena@uniroma1.it (D.M.); francesca.orsini@uniroma1.it (F.O.); felicemarco.damato@uniroma1.it (F.M.D.); paola.frati@uniroma1.it (P.F.); raffa.rinaldi@uniroma1.it (R.R.); 2 Research Unit of Bioethics and Humanities, Campus Biomedico University of Rome, 00128 Rome, Italy; v.tambone@unicampus.it; 3 Department of Psychiatry, McGill University, Montreal, QC H4A 1A1, Canada; giuseppe.dandrea6@studio.unibo.it; 4 Donders Institute for Brain, Cognition and Behaviour, Radboud University, 6500 HB Nijmegen, The Netherlands; drgiuseppefanelli@gmail.com; 5 Department of Human Genetics, Radboud University Medical Center, 6500 HB Nijmegen, The Netherlands; 6 Department of Biomedical and Neuromotor Sciences, University of Bologna, 40123 Bologna, Italy; 7 Forensic Psychiatry Unit, Department of Psychiatry, Bakirkoy Prof Mazhar Osman Training and Research Hospital for Psychiatry, Neurology, and Neurosurgery, 34147 Istanbul, Turkey; yhasanbalcioglu@gmail.com; 8 Department of Psychiatry, University of Oxford, Oxford OX3 7JX, UK; howard.ryland@psych.ox.ac.uk; 9 Oxford Health NHS Foundation Trust, Oxford OX4 4XN, UK; 10 U1172—Lille Neuroscience & Cognition (LilNCog), University of Lille, Inserm, CHU Lille, 59000 Lille, France; thomas.fovet@chu-lille.fr; 11 Clinic for Forensic Psychiatry, University Medicine Rostock, 18147 Rostock, Germany; birgit.voellm@med.uni-rostock.de; 12 Department of Psychiatry, University of Santiago de Compostela (USC), 15706 Santiago de Compostela, A Coruña, Spain; franciscojavier.vicente@usc.es; 13 Psychiatry Service, Vigo Health Area, 36211 Vigo, Pontevedra, Spain; 14 Division of Psychiatry and the Law, University of California Davis Medical Center, University of California, Sacramento, CA 95817, USA; clscott@health.ucdavis.edu

**Keywords:** mental disorders, commitment of mentally Ill, forensic psychiatry, community mental health services, risk assessment, health care reform, legislation, mental health, community treatment orders, Italian psychiatric system, ethics in involuntary psychiatry

## Abstract

**Background**: This article presents an international comparative review of involuntary psychiatric care, Community Treatment Orders (CTOs), and forensic mental health services, with operational implications for Italy. Italy has a community-based model inspired by the “Basaglia Law” (Law No. 180/1978), emphasizing deinstitutionalization and continuity of care. Nevertheless, risk governance gaps persist for high-complexity patients, imposing a disproportionate legal and clinical burden on mental health professionals. This group includes individuals who refuse treatment despite meeting criteria for compulsory admission, patients at elevated risk with substantial management complexity, and offenders with a current or suspected psychiatric disorder. **Methods**: We conducted a comparative legal and policy review across seven jurisdictions (Italy, England and Wales (UK), France, Germany, Spain, the United States, and Canada) to map frameworks for involuntary treatment, forensic services, CTOs (or equivalents), and community-based risk management. We also extracted procedural safeguards, duration and renewal limits, and interfaces with forensic services. **Results**: CTOs are available in five of the seven jurisdictions (England and Wales, France, Spain, the United States, and Canada) but are absent in Italy and Germany. We propose a three-pillar framework: (1) enforceable outpatient measures, including CTOs; (2) Forensic Psychiatry Units within Local Health Authorities; and (3) oversight boards with judicial, clinical, and social representatives. These components aim to redistribute responsibility, ensure continuity of care, and provide proportional oversight within a least restrictive, graduated system. **Conclusions**: When narrowly targeted, time limited, and paired with robust safeguards and service-quality standards, CTOs can support adherence and continuity for patients who repeatedly disengage from care. For Italy, integrating this instrument within the three-pillar framework and under independent oversight could strengthen patient rights and public safety, reduce revolving-door admissions, and improve outcomes.

## 1. Introduction

Law 180/1978, also known as the “Basaglia Law”, marked a historic turning point in Italian mental health policy [1]. It ordered the closure of the psychiatric asylums and promoted a community-based care model grounded in human rights and dignity [2]. This approach was unique on the international stage and established Italy as a leading example of non-institutionalizing psychiatry centered on the therapeutic relationship and social inclusion of patients [3].

Nearly fifty years after its approval, new challenges have emerged that the original statute could not foresee; rising comorbidity with substance use disorders [4,5], erosion of family support networks [6], substantial migration flow [7], a chronic shortage of intermediate facilities between hospital and community care [8], increased clinical and social complexity of service users [9,10], and a growing prevalence of treatment-resistant conditions [11]. Structural shortages in funding, staffing, and inter-agency protocols between health and legal systems compel the psychiatric governance model to mediate between reformist ambitions and the pragmatic limitations of daily management [12]. Mental health professionals face high clinical and social complexity with limited regulatory and organizational support. They continuously encounter ethical dilemmas [13,14,15,16], medico-legal risks [17], and heavy workloads, which fuel chronic stress, burnout, and loss of professional commitment [18,19]. According to international evidence, psychiatrist burnout often stems from patient violence or suicide, chronic under-resourcing, professional isolation, and ill-defined roles that carry responsibility without commensurate authority [20,21]. These pressures are amplified in public services where clinical demand meets structural deficits and regulatory ambiguity. Italian surveys confirm high levels of emotional exhaustion and depersonalization among practicing psychiatrists, as well as low job satisfaction [22,23]. Additionally, moderate burnout has been found among trainees, which has been linked to workload and organizational dysfunction [24]. The unintended bureaucratization of the Basaglia reform (understood as the fragmentation of responsibilities, excessive procedural formalism, and the absence of responsive, community-based risk-management mechanisms) has further limited clinical autonomy, often leaving psychiatrists to manage high-complexity patients largely on their own. The resulting “double bind” of protecting liberty while ensuring safety without intermediate mechanisms heightens legal vulnerability and feelings of powerlessness [25,26]. Fear of litigation, lack of institutional protection, and perceived erosion of clinical efficacy contribute to a vocational crisis evidenced by transfers to private practice, early retirement, defensive medicine, and, in extreme cases, disciplinary action or resignation [26,27,28,29].

Although the Italian mental health system is still internationally recognized for its community-based orientation [30], it must adapt to significant social, epidemiological, and regulatory transformations. Highly complex cases increasingly reflect severe psychopathology, social vulnerability, poor adherence, and risk-related disengagement [26]. In light of these critical challenges, this paper provides a thorough analysis of managing complex psychiatric cases (Table 1). The analysis focuses on three high-risk groups: (1) patients who refuse treatment, (2) individuals with psychiatric conditions posing a potential risk of auto- or hetero-aggression, and (3) forensic patients with diagnosed or suspected mental disorders. On this basis, we put forward targeted proposals to improve the effectiveness and equity of the current system. To our knowledge, this is the first cross-jurisdictional synthesis to explicitly bridge civil and forensic risk governance streams across seven systems. The scarcity and fragmentation of comparable legal, policy, and service information further justify a comparative analysis for the management of high-complexity psychiatric cases. Building on this mapping, we propose an operational three-pillar framework for Italy and a concrete, safeguarded model of more structured outpatient treatment.

## 2. Materials and Methods

This study adopts a comparative medico-legal approach to analyze national frameworks for involuntary treatment, forensic psychiatry, and risk governance in seven countries: Italy, England and Wales, France, Germany, Spain, the United States, and Canada. Sources include statutory regulations, mental health policy documents, and the peer-reviewed literature indexed in PubMed, PsycINFO, and Scopus. A detailed description of inclusion and exclusion criteria, search strategy, and selection process is provided in Supplementary Materials (File S1).

We analyzed each country profile in relation to three high-complexity populations outlined in the background section: (1) individuals who refuse treatment but require involuntary admission; (2) non-offending patients with mental disorders associated with a significant risk of self-harm or violence toward others; and (3) offenders with confirmed or suspected mental disorders. For each group, we assessed eligibility criteria, procedural safeguards, and care pathways.

These findings informed the development of a three-pillar care-and-risk governance framework to improve continuity of care, proportionate legal oversight, and shared institutional responsibility in Italy.

## 3. Results

The following section presents the results of a cross-national analysis of seven countries, focusing on legal and institutional responses to three high-complexity psychiatric profiles. Each jurisdiction is examined based on its legislative framework, the availability of community-based compulsory measures, and the organization of forensic psychiatric care. This comparative overview informs the rationale behind the proposed three-pillar governance model for Italy. Table 2 summarizes the key elements of involuntary admission and step-down pathways, and Table 3 provides an overview of forensic psychiatric measures across the studied jurisdictions.

### 3.1. Italy

Italy’s mental health system is founded on Law 833/1978 (which integrated Law 180/1978) and its implementing Decree 309/1990. On paper, the Italian model of involuntary psychiatric care is exemplary: any compulsory intervention (formally known as “Trattamento Sanitario Obbligatorio—TSO”) must pass a three-tier procedural filter—two independent medical or psychiatric certificates, a mayoral decree, and judicial validation by the Tutelary Judge (“Giudice Tutelare”) within forty-eight hours. The initial detention period lasts seven days and may be renewed on a weekly basis with adequate clinical justification.

During TSO, treatment—including psychotropic medication and electroconvulsive therapy—may be administered without consent, provided it is proportionate and adequately documented. TSO may only be implemented in the locked psychiatric wards of general hospitals (“Servizi Psichiatrici di Diagnosi e Cura—SPDC”). Intermediate facilities are legally excluded, resulting in an all-or-nothing system with no options for graduated risk management. In practice, the very procedural rigor and lack of intermediate structures that once distinguished the Italian model now expose systemic weaknesses. The country lacks medium-security units, forcing SPDCs to house patients whose risk profiles would otherwise warrant specialized secure facilities.

Furthermore, in its recent judgment No. 76/2025, the Constitutional Court ruled that the current TSO framework is unconstitutional insofar as it fails to guarantee the direct hearing of the individual concerned and the formal notification of the decree. The Court reaffirmed the procedural obligation to ensure the effective participation of the patient or their legal representative, as well as the timely delivery of the measure. Non-compliance with these safeguards undermines the legal validity of the intervention and raises serious concerns regarding the protection of fundamental rights.

In cases of persistent risk in the absence of a criminal conviction, patients remain in SPDCs under restrictive conditions (the TSO is renewed every seven days). Transfers often involve police escorts, and the prolonged use of mechanical restraints should be reported to both the Tutelary Judge and the Regional Health Authority.

In some Italian regions, a form of compulsory outpatient treatment—commonly referred to as “TSO extraospedaliero”—has been formally regulated through local protocols, based on an expansive interpretation of Articles 33 and 34 of Law 833/1978. Although legally permitted within the national framework, its use remains sporadic and lacks uniform procedural standards. Unlike the standard inpatient TSO, this measure does not require judicial validation [31]. In practice, it often consists of the forced administration of long-acting antipsychotic medication, typically delivered in community settings such as mental health centers or even at the patient’s home. The absence of judicial oversight and minimal procedural safeguards raise serious ethical and legal concerns, particularly regarding the protection of patients’ rights and the risk of discretionary enforcement.

For offenders found not criminally responsible by reason of mental disorder, Decree-Law 81/2014 introduced a major structural reform by closing forensic psychiatric hospitals and establishing a decentralized network of small, community-based Residences for the Execution of Security Measures (REMS) [32,33,34]. Since that 2014 reform, no intermediate-security infrastructure beyond REMS has been created to relieve SPDCs of forensic cases. When courts find offenders criminally irresponsible (or partially so) they order placement in an REMS, each facility hosting no more than twenty residents. The duration of placement cannot exceed the statutory maximum for the index offense and is subject to annual judicial review. With just 0.9 forensic beds per 100,000 inhabitants (versus 23.3 in Belgium), Italy now has one of the lowest rates of secure psychiatric provision in Europe [35,36]. As a result, law enforcement is often asked to compensate for system shortfalls. The severe shortage of beds in REMS facilities has created lengthy waiting lists, forcing courts to detain affected individuals in ordinary prisons, SPDCs, or even at home until a placement becomes available.

In the absence of intermediate-security forensic units or supervised non-custodial alternatives, magistrates are faced with an untenable “all-or-nothing choice”—prison, home, or clinically inappropriate hospitalization in general psychiatry wards—that compromises both clinical objectives and fundamental rights, a situation repeatedly censured by international monitoring bodies [37].

### 3.2. England and Wales

The legislative framework for involuntary psychiatric care in England and Wales is codified by the Mental Health Act 1983 (as amended in 2007), with procedural guidance provided by the MHA Code of Practice 2015 [38,39,40]. Under this statute, provisioned compulsion powers are delineated into “Sections”, each establishing precise clinical thresholds, maximum durations, renewal criteria, and procedural safeguards. Under “Section 2”, a patient may be lawfully detained for psychiatric assessment for up to 28 days. Should risk to self or others, or ongoing therapeutic need, persist, “Section 3” authorizes admission for treatment for up to six months, renewable [41]. During this compulsory admission, pharmacological interventions may be administered without consent; however, any continuation beyond three months (or administration of electroconvulsive therapy or neurosurgical procedures) mandates independent approval by a “Second Opinion Appointed Doctor (SOAD)” [42]. Key strengths of the system include its two-stage admission process, SOAD oversight for high-risk interventions, and a stratified secure estate composed of high-, medium-, and low-security units. In addition to these civil compulsion mechanisms, emergency powers comprise “section 4” (admission for up to 72 h on a single medical recommendation) and the inpatient holding powers under “sections 5(2) and 5(4)” [43]. Under “section 5(2)”, a doctor or approved clinician may hold an informal inpatient for up to 72 h; under “section 5(4)”, a registered mental health or learning disability nurse may hold an inpatient for up to 6 h to allow assessment under “section 5(2)”. “Sections 135–136” authorize police to convey a person to a designated place of safety for a mental health assessment as soon as practicable, for up to 24 h (extendable by 12) [44,45]. Depending on the assessment, outcomes include voluntary admission, detention under “section 2 or 3”, or discharge with or without community follow-up; where admission is indicated, transfer to a suitably secure psychiatric unit follows.

For community management after discharge, the Act provides for Guardianship Orders and Community Treatment Orders (CTOs). Guardianship (“Section 7”) allows a guardian, typically a local authority, to supervise the patient’s residence and daily activities. It does not authorize forced treatment but ensures oversight in the community [39]. CTOs (“Section 17A”), introduced in 2007, allow clinicians to impose a treatment plan as a condition for discharge. In the event of non-compliance, the patient may be recalled to the hospital for up to 72 h [46,47]. CTOs serve as a legal bridge between inpatient care and community supervision; however, their effectiveness remains uncertain. The OCTET trial and subsequent analyses found no reduction in readmissions or improvements in clinical or social outcomes compared with “Section 17 leave” [48].

In the forensic context, compulsory care for mentally disordered offenders is governed by Part III of the Mental Health Act 1983. Courts may substitute custody with a Hospital Order (“Section 37”) and, where necessary for public protection, add a Restriction Order (“Section 41”), which places leave, transfer, and discharge under Ministry of Justice oversight. The Act also provides for transfer from prison to hospital: “Section 47” for sentenced prisoners and “Section 48” for those on remand. In either case, a Restriction Direction (“Section 49”) may be added, which has the same effect as a “Section 41” restriction. For completeness, the Crime (Sentences) Act 1997 also introduced the hospital and limitation direction (“Section 45A”), a “hybrid” order combining a custodial sentence with an immediate hospital direction [49].

In cases where an offender presents a significant risk to public safety, a Restriction Order pursuant to “Section 41” of the Mental Health Act may be imposed alongside a Hospital Order (“Section 37”), placing leave, transfer, and discharge under Ministry of Justice (MoJ) oversight and rendering psychiatric detention indeterminate, subject to periodic forensic risk reviews. In addition, for restricted patients (“Sections 37/41” or “47/49”), the court or the First-tier Tribunal may direct “conditional discharge”: the patient leaves hospital under clearly defined conditions, remains “liable to recall” if risk escalates or conditions are breached, and is supervised in the community (sometimes colloquially described as a “CTO with teeth”). The legislation provides procedural safeguards; however, the nearest relative has more limited powers under “Section 37” than under the civil sections: they may apply to the “First-tier Tribunal” for discharge (whereas under “Sections 2/3” they may apply directly for discharge, subject to a barring certificate by the responsible clinician). For patients subject to a Restriction Order, any discharge also requires MoJ approval, typically with responsible clinician support. While comprehensive, this framework continues to prompt debate over the balance between patient autonomy and public protection, particularly in view of prolonged detentions in “secure wards” and the ethical issues inherent in compulsory treatment in the community [50,51].

### 3.3. France

In France, involuntary psychiatric care is regulated by the Public Health Code. The most recent legislation governing its procedures is the *Law of 5 July 2011*, amended by the *Law of 27 September 2013*. Currently, there are two main pathways to involuntary admission [52,53].

On the one hand, involuntary psychiatric care at the request of a third party may be initiated upon receipt of a written application from a qualified third party (usually a relative or a trusted individual) (“*Soins psychiatriques à la demande d’un tiers*”, SPDT). In this case, hospitalization is ordered by the hospital director. This request must be supported by two independent medical certificates confirming that (1) the patient’s mental state requires immediate care with constant or regular medical supervision; and (2) the mental disorder makes the patient unable to consent (Art. L3212-1 of the Public Health Code). In urgent cases where the patient’s integrity is at serious risk, only one certificate is required (“*SPDT en urgence*”, Art. L3212-3). When there is imminent danger to the patient and no third party is available, a single medical certificate is sufficient to initiate care (“*Soins psychiatriques en péril imminent*”, SPPI, Art. L3212-1-II-2) [54].

On the other hand, admission may also be ordered by the local state’s representative *(“Préfet”)*. In this case, the *“Préfet”* has the authority to order immediate admission based on a single medical certificate in situations of psychiatric disorders requiring care and involving the disturbance of public order or threats to the safety of others (“*Soins psychiatriques sur décision du* R*eprésentant de l’État*”, Art. L3213-1) [55]. Individuals declared not criminally responsible due to a mental disorder by a criminal court are generally referred to a psychiatric hospital under an SPDRE procedure (*Penal Code*, Art. 122-1). Under these circumstances, discharge requires a psychiatric evaluation by two independent psychiatric experts [56].

For all involuntary psychiatric care measures (SPDT, SPPI, SPDRE), patients must undergo a mandatory 72 h observation period. During this time, two medical certificates are required: one within 24 h of admission and another within 72 h of admission. An additional certificate must then be issued between the sixth and eighth day of hospitalization. The measure must also be validated by a liberty and custody judge (“*Juge des libertés et de la detention*”, JLD) before the twelfth day, and is thereafter subject to judicial review every six months [57].

Compulsory Community Treatment (CCT) can be initiated at the end of a full-time involuntary psychiatric hospitalization (“*Programmes de soins”*). This involuntary outpatient treatment can only be decided by the treating psychiatrist, without the need for immediate referral to the JLD, and is not court-ordered. There is no minimum or maximum duration for this care. If the person does not comply with the ambulatory program, they may be readmitted to the hospital.

In the criminal justice context, courts may impose post-custodial preventive measures. These measures range from court-ordered care—primarily psychiatric or psychological treatment after the individual has served their prison sentence—to preventive detention (“*rétention de sûreté*”), a renewable custodial measure of up to two years [58,59].

There are additional regulatory frameworks for high-security units such as “unités pour malades difficiles (UMD)” [60], civil forensic centers for patients deemed at high-risk of violence, and “*unités hospitalières spécialement aménagées* (UHSA)”, full-time psychiatric hospitalization units exclusively for incarcerated people [61].

This layered model, combining legal oversight with clinical stratification, aims to reconcile individual rights, due process, public protection, and therapeutic continuity. Nevertheless, debate continues as to whether a broader use of less-restrictive pathways actually reduces involuntary admissions or simply shifts the burden elsewhere [62].

### 3.4. Germany

In Germany, involuntary psychiatric care is regulated by each Land’s “Psychisch-Kranken-Gesetz (PsychKG)” and the federal “Strafgesetzbuch (StGB §§ 20–21, 63–66)” [63,64,65]. This decentralized structure leads to some regional variation. The legal framework distinguishes public law detention (PsychKG) from civil law guardianship measures under the Civil Code and strictly separates detention from medical treatment. Several Länder also provide visiting commissions (Besuchskommissionen) for inspection and oversight.

Three legal pathways authorize involuntary detention:

(1) Criminal commitment (§§ 63–66 StGB): Individuals found not criminally responsible due to mental disorder, profoundly reduced consciousness, intellectual disability, or any other serious mental abnormality (§ 20) may be hospitalized indefinitely under § 63 if there is a risk of further offending. Those with diminished responsibility (§ 21) may receive a reduced sentence yet may still be detained if assessed as dangerous. Preventive detention under § 66 targets offenders deemed dangerous despite full responsibility. §64 allows for the detention of individuals who have committed an offense due to a serious substance use disorder. Such detention does not require diminished criminal responsibility, and individuals receive a parallel prison sentence. They may be returned to prison if treatment is deemed unsuccessful and the time of detention is limited to two years plus two-thirds of the parallel prison sentence [66].

(2) Guardianship-based detention (BGB § 1906; now §§ 1831–1832): People with severe mental illness and a risk to themselves or others may be hospitalized upon an application by a guardian to the court. Since 2013, courts must also verify incapacity, medical necessity, absence of alternatives, and prior patient information, supported by an external psychiatric assessment, and limit treatment orders to two weeks initially.

(3) Emergency detention (PsychKG): In acute crises, police or doctors may initiate hospitalization, which must be confirmed within 24 h by a Local Court. Continued detention requires regular reassessment and judicial renewal. These laws apply only to inpatient care in cases of immediate danger.

Outpatient coercive treatment (e.g., Community Treatment Orders) is currently not permitted under German law; coercive psychiatric measures occur exclusively in inpatient settings [36].

However, in November 2024, the Federal Constitutional Court held that the blanket statutory requirement that coercive medical treatment be carried out only in hospital is partly unconstitutional and instructed the legislature to adopt a constitution-compliant framework by the end of 2026; until then, the existing provision continues to apply, subject to constitutional limits.

The law is very strict on involuntary pharmacological treatment, which is only allowed (except in life-threatening circumstances) with a second opinion and a court order from the outset. Mechanical restraint is used in Germany but, again, a court order is required if the measure exceeds 30 min [67,68].

The system’s strengths include judicial oversight, independent psychiatric review, and procedural safeguards. Families have no formal authority but may petition for hospitalization or be appointed as guardians.

For high-complexity individuals without criminal charges, inpatient care proceeds on a voluntary basis where accepted; only where the person refuses and the statutory criteria are met may a court order compulsory admission. For those with criminal convictions or offending behavior, courts may impose mandatory aftercare for up to five years, with conditions such as abstaining from drug use, attending scheduled appointments, and residing at a specified address. Pharmacological treatment is not ordinarily encompassed within this form of supervision and would require a separate legal basis [69]. Statistical data reveal a consistent rise in court-ordered admissions [70]. Between 2000 and 2011, cases under § 1906 BGB (now §§ 1831–1832) increased by 67% (from 87,606 to 146,190), and PsychKG admissions rose by 37% (from 57,051 to 78,147), against a 23% overall increase in psychiatric hospitalizations. Detention rates of mentally disordered offenders have also increased, particularly those under §64 StGB. Current concerns include legal uncertainty over coercive somatic treatment, rising detention rates, and the effects of hospital funding reforms. Germany’s system strives to balance protection, rights, and therapeutic goals, but regional disparities remain [70].

### 3.5. Spain

In Spain, involuntary admission is governed by Article 763 of the “Ley de Enjuiciamiento Civil (LEC)” for civil law, Articles 95–108 of the “Código Penal”, and “Ley Orgánica” 1/2015 for criminal law. In the civil law context, “internamiento” refers to the compulsory hospitalization of individuals with severe mental disorders who pose a serious danger to themselves or others, where less restrictive measures have failed, prove insufficient, or are unfeasible, and where the patient does not consent [71,72]. Article 763 LEC (under which civil involuntary hospitalization must be medically indicated, serve a defined therapeutic objective, and never be used for social or punitive purposes) establishes two distinct civil pathways:

(1) Ordinary civil “internamiento” (non-urgent). This pathway is invoked when the patient refuses hospitalization in the absence of an acute clinical emergency. Transfer to a psychiatric facility is permissible only after a judge has issued explicit prior authorization [71].

(2) Urgent civil “internamiento”. This mechanism may be activated by any third party, irrespective of familial or prosecutorial ties, who brings a presumptively severely mentally disordered individual to a psychiatric service. The facility must report the admission to the guardianship judge within 24 h, and the judge, after hearing the patient, the Public Prosecutor, the attending psychiatrist, and any other relevant parties, must confirm or revoke the detention within 72 h [71]. In addition, individuals subject to involuntary admission may seek habeas corpus (Const. Art. 17(4); Organic Law 6/1984), an emergency judicial remedy that ensures prompt review of the lawfulness of detention and, where appropriate, immediate release; the court must issue a decision within 24 h.

Spanish legislation sets no predefined maximum duration for civil “internamiento”. The measure must persist only for the period strictly required by the proportionality principle affirmed by the Constitutional Court [72]. From a clinical standpoint, compulsory admission is reserved for acute decompensation of substantial severity in which outpatient management is impracticable. Treating psychiatrists are obliged to submit a clinical report to the court at least every six months. However, certain Autonomous Communities, such as Catalonia, demand more frequent judicial review (every two months). Discharge may be ordered unilaterally by the treating psychiatrist and need only be communicated to the court; no additional judicial mandate is necessary. The statutory scheme does not require proof of dangerousness. This reflects the medical (not penal) nature of the measure: admission aims to enable treatment in a controlled environment for individuals with a serious mental disorder whose prognosis would worsen without care, who are likely to improve with treatment, and who lack insight into their illness [73,74]. Psychosis and treatment refusal are common grounds for emergency admissions [75]. Where dangerousness is not established, courts may order non-custodial alternatives such as residence restrictions, periodic judicial review, electronic monitoring, and, where available, involuntary outpatient treatment, rather than authorizing hospital detention. Conversely, certain protected groups may be admitted involuntarily on protective grounds irrespective of dangerousness, notably minors and adults under guardianship, for whom the guardian’s consent alone is insufficient and judicial authorization is required. Spain remains without a nationwide statute governing compulsory care in the community of involuntary outpatient treatment (IOT). Repeated parliamentary initiatives did not secure passage, leaving the legal foundation for IOT fragmented and dependent on the general provisions of Article 763 LEC [76]. In the absence of primary law regulation, several provincial courts have developed ad hoc programs. The most mature scheme operates in Valencia, where the Civil Court has issued approximately 140 IOT orders over about ten years since 2003, the great majority involving schizophrenia-spectrum disorders [76]. Smaller caseloads have been documented in San Sebastián, Barcelona, Gijón, and Murcia, reflecting a permissive interpretation of Article 763 that treats IOT as the least restrictive alternative to inpatient commitment [76]. Empirical evidence remains equivocal. Observational cohorts suggest reductions in rehospitalization and emergency utilization, whereas controlled studies yield inconsistent effects on clinical stability and service use [59]. Nevertheless, professional support is strong: a 2020 survey of Spanish psychiatrists reported that 92.8% favor specific legislation for IOT, and 78.6% consider the measure beneficial rather than stigmatizing [77].

For criminal offenders, the “Código Penal” (Articles 101–103) provides for “internamiento” in psychiatric, detoxification, or special educational facilities when the defendant is deemed not to be imputable due to a severe mental disorder (“alteración psíquica grave”), complete intoxication, or persistent perceptual disturbances. These security measures, renewable annually, may exceed the penal maximum by up to five years and are often converted to outpatient treatment as the risk decreases; the time spent in “internamiento” is deducted from any residual sentence [78].

“Ley Orgánica” 1/2015 confirmed this dual therapeutic–custodial model. When a mental disorder emerges after sentencing, the Penitentiary Surveillance Judge (“Juez de Vigilancia Penitenciaria”) may suspend the execution of the sentence and order an appropriate medico-legal “internamiento”. In practice, most inmates with severe mental illness are held in ordinary prisons, where psychiatric care is limited, fragmented, and reliant on part-time consultants. Only two forensic psychiatric hospitals (in Alicante and Seville) serve the not-imputable population under the Ministry of Interior. Due to capacity and staffing deficits, many individuals with severe psychiatric conditions remain in general prison modules lacking appropriate infrastructure.

Although a 2003 law enabled the regionalization of prison healthcare, only Catalonia, the Basque Country, and Navarra completed the integration into their public health systems. These regions offer more structured and continuous psychiatric care through dedicated prison mental health units and coordinated reintegration programs. Basque and Catalan models are among the most developed, featuring internal psychiatric services (e.g., Psychiatric Hospitalization Internal Rehabilitation Unit Closed—PHIRU-C) and individualized discharge pathways [75].

### 3.6. Canada

Canada is a federal state composed of ten provinces and three territories, each of which is responsible for its health legislation and services. This has resulted in thirteen distinct Mental Health Acts, which, while sharing core principles, such as the favoring of voluntary admission, exhibit clinically significant differences [79]. Most jurisdictions are governed by common law, whereas Québec follows a civil code system akin to that of France. All provincial laws must conform to the Canadian Charter of Rights and Freedoms, which serves as the country’s constitutional framework. For involuntary admission or compulsory inpatient or community treatment to be valid, three components must be satisfied: procedure, committal criteria, and rights safeguards [80].

Involuntary psychiatric intervention is governed jointly by the provincial Mental Health Acts, section 672.1–672.95 of the federal Criminal Code (which regulates individuals found Not Criminally Responsible on account of Mental Disorder—NCRMD), and the Canadian Charter of Rights and Freedoms. This tripartite framework embodies the principles of collegial review through “Mental Health Review Boards”, the “least restrictive alternative,” and mandatory rights advice. However, it also faces challenges of interprovincial heterogeneity and the over-representation of Indigenous peoples in compulsion statistics [80].

The statutory pathway unfolds in a series of graduated phases. First, an “emergency hold” or apprehension may be effected by police officers or designated healthcare professionals for up to 24 h in Alberta, 48 h in British Columbia, and 72 h in Ontario and Québec. If, at the end of that period, the individual is still assessed as posing a serious risk to self or others, a second psychiatrist issues a certificate of involuntary admission contingent on independent concurrence. In most provinces, this medical certification is sufficient; however, in Québec, ongoing detention requires court authorization; in New Brunswick, ongoing detention proceeds by medical certification with review mechanisms available. Should the criteria persist, successive renewals follow in Ontario for sequential periods of 30, 60, and 90 days; in British Columbia initially for one month, then three months, and thereafter in successive six-month periods; and in Québec by court authorization with a clinical report at 21 days and subsequent reviews at intervals of no less than three months—each requiring contemporaneous clinical–legal reassessment and, where applicable, a hearing before a Mental Health Review Board to confirm ongoing risk [81]. Upon involuntary admission, patients must also be promptly informed of their rights, including the grounds for detention, the right to legal counsel, and the possibility to appeal before a Review Board.

Most jurisdictions transition stabilized patients to the community under a renewable six-month Community Treatment Order (CTO), binding them to an individualized plan of treatment and supervision and authorizing police recall for non-adherence. As of 2019, CTOs or equivalent mechanisms are available in nine of the thirteen Canadian jurisdictions [82]. These are typically reserved for individuals with a documented history of psychiatric hospitalization (e.g., three or more involuntary admissions or at least 60 inpatient days in the past two years in Newfoundland and Labrador). Unlike many provinces, Alberta’s CTO criteria do not invariably require prior hospitalization. All CTOs require that the necessary community services be available and that a formal treatment plan, often involving family or support networks, be in place [82].

Provincial legislation on treatment rights varies markedly. In Ontario and several other provinces, capable patients, even if involuntarily detained, retain the right to refuse psychiatric treatment, and substitute decision-makers cannot override an advance directive unless specific conditions are met. Conversely, in provinces such as British Columbia, the medical director of the facility may authorize treatment without consent, viewing the state’s duty to restore liberty as overriding individual refusal. This reflects a broader structural distinction across jurisdictions: in the “state” model (e.g., British Columbia), an institutional appointee (such as the hospital director or a quasi-judicial board) authorizes treatment for patients who are incapable. In contrast, the “private” model (e.g., Ontario) entrusts decision-making to a substitute decision-maker, usually a relative, who is bound to follow any applicable advance directive unless doing so would endanger the patient or others [83].

When an accused is found “Not Criminally Responsible because of Mental Disorder” (NCRMD)” under the Criminal Code, a Provincial Review Board conducts a disposition hearing to order detention, conditional discharge, or absolute discharge, with annual risk-based reviews mandated. Throughout judicial or quasi-judicial oversight, repeated clinical evaluation and the least-restrictive principle aim to balance civil liberties, therapeutic necessity, and public safety imperatives [84,85].

### 3.7. United States

In the United States, involuntary psychiatric hospital commitment is governed by state mental health statutes and shaped by two important landmark United States Supreme Court rulings. First, in *O’Connor v. Donaldson* (1975), the Court held that involuntarily committing a non-dangerous mentally ill person who can safely survive in the community violates their substantive due process right to liberty. Second, the ruling in *Addington v. Texas* (1979) raised the standard of proof for involuntary psychiatric commitment from “preponderance of evidence” (e.g., more than 50% certainty) to “clear and convincing evidence” (e.g., more than 75% certainty).

All fifty states authorize an initial emergency psychiatric hold (typically 24–72 h), during which the individual typically receives written notification of their rights and a prompt psychiatric evaluation [86,87,88]. Common risk criteria for involuntary detainment include danger to self, danger to others, or that the person is gravely disabled as a result of their mental illness. If at least one of these risk criteria persists after the initial emergency hold, a short-term civil commitment order may be issued, which will be adjudicated at a probable-cause hearing within 5–7 days. This hearing is a preliminary judicial review specific to the U.S. legal system intended to determine whether a clinically and factually grounded justification exists for continuing involuntary psychiatric treatment. The continued commitment is typically limited to about 14 days pending a full hearing. A long-term commitment (often 90 days initially, extendable to 180 days or more) requires judicial authorization, a finding of grave disability or danger to self or others, and the absence of a less restrictive alternative [81].

Over the past two decades, many states have added a community-based tier via Assisted Outpatient Treatment (AOT): six- to twelve-month court mandates requiring medication adherence, substance use counseling, and case management, with non-compliance triggering brief rehospitalization. AOT eligibility varies widely and is often reserved for individuals with repeated hospitalizations or clear deterioration despite available voluntary care [71,72].

Forensic pathways for involuntary commitment in the U.S. include “Not Guilty by Reason of Insanity” (NGRI) verdicts, which entail commitment to state forensic hospitals until the individual demonstrates that they no longer pose a danger to others [89,90]. In contrast, “Guilty But Mentally Ill” (GBMI) designations impose penal custody combined with psychiatric treatment, without exempting the defendant from criminal responsibility or altering the sentence duration [91,92,93].

Despite formal procedural safeguards, such as legal counsel, periodic reviews, and judicial oversight, the U.S. system remains marked by interstate heterogeneity, limited forensic hospital capacity, and a persistent revolving-door dynamic [94]. Involuntary outpatient and inpatient commitment continue to spark ethical debate [95], especially concerning coercion thresholds, patients’ right to refuse treatment, and post-sentence civil commitment of high-risk individuals under Sexually Violent Predator laws [96].

### 3.8. International Comparative Studies

Four comparative contributions have significantly enriched our understanding of the substantial heterogeneity that characterizes involuntary and forensic psychiatric care across national contexts.

Dressing and Salize provided one of the earliest systematic overviews of mental health legislation across European Union Member States, highlighting pronounced procedural variability with minimal correlation to actual rates of compulsory admission [97].

Zhang et al. extended the scope globally, encompassing jurisdictions from Europe, North and South America, Asia, and Oceania, and compared admission criteria and legal safeguards across more than a dozen countries [81]. Their findings underscore the influence of cultural traditions, normative frameworks, and ethical tensions on the use of coercion in mental health systems.

Sheridan Rains et al. conducted an epidemiological study across 22 countries in Europe and the broader Commonwealth area, comparing annual incidence rates of involuntary hospitalization with legislative, demographic, economic, and service-related variables to identify systemic determinants of coercive psychiatric practices [98].

Finally, Salize et al. examined the structure and capacity of forensic psychiatric services across 24 European Union Member States, revealing significant disparities in legal definitions, admission criteria, detention duration, and institutional resources. Their findings call for the establishment of minimum common standards at the European level [35].

Taken together, these studies delineate a fragmented and uneven landscape of coercive and forensic psychiatric governance, marked by tensions between public health imperatives, procedural safeguards, and the protection of individual rights.

## 4. Discussion

The comparative analysis of involuntary psychiatric admission systems that we conducted, enriched by the contributions of Dressing and Salize, Zhang et al., and Sheridan Rains et al., confirms the substantial legal, procedural, and cultural heterogeneity across international jurisdictions [81,97,98].

### 4.1. Involuntary Care for Non-Offending Patients

In most Central and Northern European countries (e.g., Germany and France), as well as in North American and Asian systems examined by Zhang et al., the predominant criterion for involuntary admission is the presence of “current risk to self or others.”

Some jurisdictions complement this with additional criteria such as marked mental deterioration or urgent clinical need for treatment. Italy, by contrast, adopts three criteria—urgent need for treatment, lack of capacity or willingness to consent, and the absence of suitable alternatives—without any explicit reference to dangerousness. While this approach appears more clinically inclusive, it introduces subjectivity and marked regional variation, echoing the concerns raised Dressing and Salize [97].

Systems with stronger procedural safeguards require prompt judicial validation (e.g., within 24 h in Germany and 48 h in Italy) and periodic reviews. However, few countries have an independent, multidisciplinary oversight body that provides medium- to long-term clinical–legal supervision. In Italy, once the Mayor and Guardianship Judge have validated the initial order, no legally binding intermediate reviews follow, leaving clinicians without structured, multilevel oversight [81,97].

A further weakness in the Italian framework is the absence of scalable measures between compulsory hospitalization (SPDC) and full discharge. The current dichotomy, “all or nothing”, offers no statutory tool comparable to Compulsory Community Treatment, which elsewhere allows mandatory outpatient care (e.g., pharmacotherapy, psychotherapy, toxicology screening).

#### Compulsory Community Treatment (CCT)

Compulsory Community Treatment (CCT)—implemented in many jurisdictions through a Community Treatment Order (CTO)—establishes adherence to a prescribed care plan as a statutory requirement for individuals with severe mental disorders who reside in the community. The measure remains the subject of vigorous debate. Proponents frame CCT as a less restrictive alternative to involuntary hospital admission, arguing that it can temper the “revolving-door” phenomenon of repeated admissions and promote continuity of care [99,100]. In contrast, clinicians, service users, and legal scholars question the legitimacy of curtailing the liberty of competent adults who have committed no offense, highlighting the potential erosion of the therapeutic alliance and the paucity of compelling evidence for effectiveness [48,101]. Some qualitative investigations nevertheless suggest that a subset of patients prefers CCT to prolonged inpatient care, and that caregivers and clinicians frequently regard it as a valuable intervention [102,103]. The apparent benefit, however, may derive principally from the statutory duty imposed on services to provide assertive follow-up rather than from a direct therapeutic effect on patient behavior [104,105].

Quantitative evidence remains inconclusive [106,107]. A recent systematic review by Barnett et al. screened 41 studies (encompassing 189,749 participants) and meta-analyzed 39, including randomized controlled trials (RCTs), observational studies with contemporaneous controls, and pre–post designs [108]. In pre–post comparisons (largely involving patients discharged after lengthy admissions), CCT was associated with a substantial reduction in readmissions (standardized mean difference [SMD] ≈ 0.80) and a moderate decrease in inpatient bed days (SMD ≈ 0.66), together with marked increases in community service utilization (SMD ≈ 0.83) and treatment adherence (SMD ≈ 2.12). Heterogeneity was, however, extreme (I^2^ > 90%). Among the 20 studies with contemporaneous control groups (including four RCTs), the effects on readmission rates and length of stay disappeared; only a moderate rise in community contacts persisted (SMD ≈ 0.38), with high heterogeneity (I^2^ ≈ 97%). Barnett et al. ascribe the significant pre–post effects to regression to the mean and natural illness maturation, noting that the more rigorous controlled analyses fail to show any hospital-use advantage, implying that, at present, CCT currently does not achieve its intended reduction in bed occupancy and instead primarily results in an expansion of community-based care delivered under coercive circumstances.

Mustafa’s accompanying commentary advocates a more nuanced interpretation [109]. He argues that the sizeable improvements observed in pre–post designs may reflect the actual impact of CCT in the very population for which it is intended, individuals with severe illness and entrenched non-adherence, who are notoriously difficult to enlist in RCTs for ethical and practical reasons. Drawing a parallel with long-acting injectable antipsychotics, he notes that RCTs conducted with already cooperative patients produced negative findings. In contrast, real-world studies demonstrated clear benefits among non-adherent patients. From this perspective, traditional randomized RCTs may not represent the optimal standard for evaluating interventions targeted at clinically complex subgroups [109].

Barnett et al. themselves acknowledge important limitations: smaller numbers of RCTs lead to a limited representativeness of study samples, a wider cross-national variation in legal frameworks and clinical criteria, and a dearth of data on demographic subgroups or on patient-centered outcomes such as quality of life and satisfaction. These shortcomings weaken the certainty of pooled estimates and leave critical questions about equity and proportionality unresolved [108].

Taken together, the available evidence does not support the widespread and indiscriminate deployment of CCT. The order should be reserved for patients with severe mental illness, a well-documented history of non-adherence, and demonstrable disengagement from services, and it should be sustained only when accompanied by measurable clinical improvement [83].

### 4.2. Offenders with Psychiatric Disorders

Special attention should also be given to the management of offenders with psychiatric disorders, who represent a particularly vulnerable and complex subgroup. Our comparative analysis revealed significant cross-national differences in how these patients are managed. In countries such as Germany, the United Kingdom, the United States, and Canada, dedicated forensic pathways exist involving placement in secure therapeutic facilities, with periodic multidisciplinary risk assessment and, where applicable, judicial or tribunal oversight. In some cases, these measures are of indeterminate duration: e.g., §63 of the German Penal Code or review-based detention under Provincial Review Boards in Canada.

In Italy, by contrast, the closure of forensic psychiatric hospitals (OPG) and the establishment of REMS marked an important conceptual and legislative shift toward a healthcare-based model. However, the practical implementation has been severely deficient: Italy currently has the lowest number of forensic psychiatric beds per capita in Europe [35,36]. This structural gap has led to several inadequate and often rights-violating outcomes, including the placement of mentally ill offenders in ordinary prisons, home confinement, referral to non-specialized therapeutic communities, or supervision by Community Mental Health Services (“Centri di Salute Mentale, CSM”) or clinically unsuitable admissions in general psychiatry wards.

Critically, these community services lack the legal authority to mandate treatment compliance in noncooperative individuals. As a result, patients with severe psychiatric disorders who have committed criminal acts may entirely reject care, escaping any form of structured therapeutic containment. They may even remain in an REMS facility without taking any medication or participating in rehabilitative activities. The absence of intermediate legal instruments and a formalized multilevel clinical–legal governance renders the Italian system both fragile and dysfunctional, ultimately undermining not only the patient’s right to care but also public safety and the credibility of judicial alternatives to incarceration.

This systemic failure was explicitly acknowledged by the Italian Constitutional Court in Sentence No. 22/2022. Based on data gathered during the preliminary investigation, the Court reported that between 670 and 750 individuals—many assessed as socially dangerous and responsible for serious, including violent, offenses—were awaiting REMS admission, with average delays exceeding ten months. The Court considered these conditions incompatible with fundamental constitutional guarantees and reflective of a broader normative and organizational gap requiring urgent and systemic reform.

## 5. Organizational Proposal: A Structured Three-Pillar Model to Bridge Clinical, Legal, and Governance Gaps

**Figure 1:**
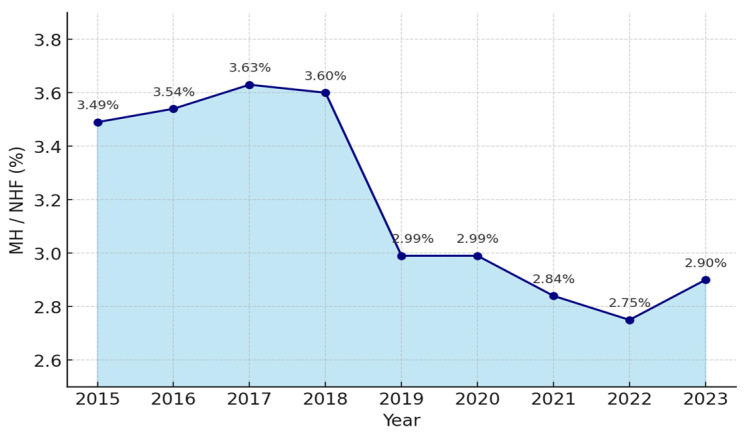
Mental health spending as % of the National Health Fund (2015–2023). MH (Mental health spending); NHF (National Health Fund); Bn (billions) [111,112,113].

Italy is in urgent need of a substantial reinvestment in psychiatric care. Over the past decade, the progressive erosion of public resources allocated to mental health services has compromised the system’s ability to innovate and remain at the forefront of clinical practice (Figure 1) [110]. Detailed data are provided in Supplementary Material Table S1.

Italy ranks among the lowest in Europe in terms of the proportion of national healthcare expenditure devoted to mental health [111]. Such funding is an indispensable prerequisite for launching structural reforms that can effectively address contemporary clinical and epidemiological challenges. The 2018–2019 inflection may reflect spending-review measures and budget re-prioritization, set against a broader context of slow, cumulative budget erosion and a progressive de-prioritization of mental health on the political agenda; see Supplementary Material Table S1.

To ease the management burden posed by high-complexity patients, currently borne almost exclusively by the Department of Mental Health, a comprehensive organizational reconfiguration is required. This reconfiguration should address the fragmentation of responsibilities and the operational isolation of individual clinicians.

Against this backdrop, we propose a three-pillar model designed to safeguard patients’ rights while simultaneously protecting public safety:

- Legal Instruments
- Forensic Psychiatry Units
- The Second Tier of Oversight and Protection: The Multidisciplinary Board

### 5.1. Legal Instruments

The authors of this work argue that Italy should pilot Community Treatment Orders on a time-limited basis. The pilot should target a narrow and clearly defined subgroup of high-complexity patients who repeatedly disengage from care and place a significant management burden on services. This position is situated within the Italian context, where compulsory admissions are brief and community tools are limited. It also recognizes that evidence from randomized trials is inconclusive, while observational studies drawn from routine practice are more favorable.

In selected cohorts, Community Treatment Orders may deliver parallel benefits. For forensic patients, they can offer a supervised community alternative, thereby preventing unnecessary court-ordered inpatient placements. For patients with poor adherence, they can strengthen linkage to community mental health services and support continuity of care. This claim rests mainly on observational evidence. With a multidisciplinary team, they can enable structured monitoring in the community and timely intervention when needed.

Any pilot should be governed by strict eligibility criteria together with periodic review by an independent Multidisciplinary Board and judicial oversight. These safeguards ensure proportionality, protect rights, and maintain continuity of care. The aim is not simply to lower readmissions. The literature on that outcome is mixed, and readmission can serve as a planned safety mechanism. The aim is to test whether a graduated legal instrument can bridge the gap between full inpatient coercion and voluntary outpatient care. The pilot should be conducted within a transparent framework with independent monitoring and prespecified evaluation, allowing for the assessment of clinical, ethical, and service-level effects before any wider adoption.

### 5.2. Forensic Psychiatry Units

Each Mental Health Department (DSM) within Italy’s Local Health Authorities (ASL) should establish specialized Forensic Psychiatry Units (FPUs) staffed by forensic psychiatrists, medico-legal experts, psychologists, and social workers.

FPUs would operate in close coordination with the Multidisciplinary Board, providing specialized assessment and follow-ups for two groups: individuals with mental illness subject to community-based judicial measures in lieu of REMS placement, and non-offender patients who present a high clinical and social risk following discharge from acute psychiatric wards (SPDCs). Their remit includes structured risk assessment, oversight of therapeutic plans delivered in custodial or community settings, and facilitation of graduated discharge pathways in collaboration with the competent public security and correctional services.

### 5.3. The Second Tier of Oversight and Protection: The Multidisciplinary Board

The Multidisciplinary Board is composed of the treating psychiatrist and, where necessary, a forensic psychiatrist from the Forensic Psychiatry Unit (FPU), the medico-legal officer of the Local Health Authority (ASL), a social worker from the Mental Health Department (DSM), a municipal representative, typically the mayor or a designated delegate, a judge from the guardianship court, and the patient, whom an independent advocate or a designated family member may accompany. This configuration ensures the integration of clinical, legal, social welfare, and experiential dimensions within a single decision-making forum.

Upon referral by the DSM or FPU, the Board must be convened promptly and, in any case, no later than three days following the request. During the joint session, following a structured and multidimensional risk assessment, the Board formulates a binding opinion. If the individual is deemed formally collaborative, the Board may approve a voluntary therapeutic and rehabilitative program under the clinical and medico-legal oversight of the FPU. Alternatively, in circumstances where substantial community supervision is warranted, the Board may issue a Community Treatment Order, thereby mandating adherence to a defined treatment plan within a non-custodial setting.

The subsequent care trajectory may include continued admission to SPDC, placement in an intermediate residential facility, transfer to a therapeutic community, or structured follow-up through community mental health services. Each intervention plan is individualized and proportionate to the patient’s clinical status, psychosocial functioning, and risk profile, typically integrating pharmacological treatment, evidence-based psychosocial therapies, and vocational rehabilitation strategies.

The Board maintains jurisdiction over individuals in the post-compulsory treatment phase, as well as over high-complexity patients referred by the FPU who are under the care of community mental health services. Its purpose is to ensure that risk containment measures are always accompanied by care pathways focused on recovery, rehabilitation, and reintegration. In the event of a serious breach of the agreed-upon therapeutic framework, the Board may issue a recall order, modeled on the English CTO, requiring a return to hospital for up to 72 h, during which the order may be revoked and the prior detention power (e.g., Section 3) reinstated following reassessment.

**Figure 2:**
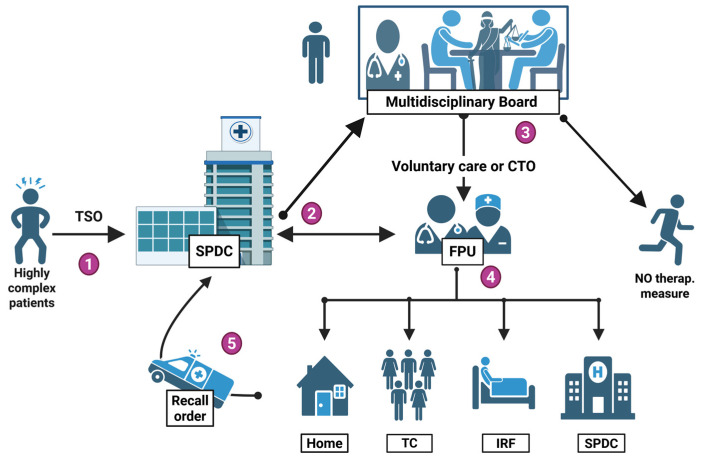
Integrated care-and-risk pathway for non-offending high-complexity psychiatric patients. ① Acute phase. A highly complex psychiatric patient is admitted to an acute psychiatric ward (SPDC) under a compulsory order (TSO). ② Referral to the Forensic Psychiatry Unit (FPU). If TSO criteria are not met—or are no longer met—yet the patient still presents a high-risk profile, the case is flagged to the FPU. The Unit also provides on-site forensic consultation to SPDC clinicians. ③ Multidisciplinary Board review. The FPU forwards the case to the Multidisciplinary Board, which meets within a few days (≤3 days). After a structured risk appraisal, the Board can offer a voluntary therapeutic–rehabilitative package under FPU supervision; issue a Community Treatment Order (CTO) for stringent supervision; or determine that no therapeutic measure (NTM) is currently indicated. ④ Implementation. The FPU operationalizes the chosen plan, delivering care at home, in a therapeutic community (TC), in an intermediate residential facility (IRF), or—if required—under continued SPDC observation. ⑤ Recall mechanism. Should the patient seriously breach the agreed plan, the Board may issue a recall order, compelling return to the hospital within 72 h for clinical and risk reassessment. Created in https://BioRender.com (accessed on 18 June 2025).

Periodic multidisciplinary reviews are conducted to evaluate treatment adherence, clinical evolution, and public safety indicators. These assessments enable dynamic adjustment of both the intensity of care and the degree of any restrictive measures in a manner that is proportionate, evidence-informed, and ethically justified. Acting in close coordination with FPUs and public security institutions, the Board serves as a transparent and legally coherent governance mechanism, upholding fundamental rights while ensuring adequate protection of the community (Figure 2).

## 6. Bioethics, Disability Law, System Limits, and Implications for Practice

### 6.1. Bioethical Considerations

Beyond its structural and organizational rationale, the proposed model is ethically grounded in a vision of care that seeks to balance autonomy and protection, as well as institutional justice and individual vulnerability. It addresses not only the issue of operational fragmentation but also the symbolic and ethical marginalization of patients whose conditions place them outside existing clinical and legal frameworks. In proposing proportionate, shared, and reviewable mechanisms of intervention, the model reinforces a relational and collaborative approach to care. Decisions are no longer made by isolated professionals but are distributed across transparent, accountable, and interdisciplinary processes. The design choices made are not only functional but also ethically motivated. These principles are becoming increasingly central to contemporary bioethics. Primarily, the model acknowledges vulnerability not as a limitation but as a condition that requires active institutional recognition and response. This perspective is rooted in the ethics of care and the concept of relational autonomy, where individuals are understood in the context of their relationships, dependencies, and social circumstances [114].

Furthermore, the emphasis on interdisciplinary collaboration and shared responsibility is rooted in the understanding that no individual professional—whether a clinician, judge, or social worker—should hold complete ethical and legal responsibility when managing complex cases. Collective decision-making has been demonstrated to distribute responsibility more fairly whilst concomitantly enhancing transparency and the quality of care.

It is also important to note that the inclusion of structured oversight and periodic review serves as an ethical safeguard. It is crucial to ensure that interventions affecting liberty and access to treatment are made in a proportionate, accountable, and revisable manner. This is to protect both patients’ fundamental rights and public trust.

From a bioethical perspective, the legitimacy of any coercive measure in psychiatry is contingent upon the establishment of a clear borderline. Coercion can only be justified as an exceptional intervention, strictly necessary to prevent imminent harm, and proportionate to the risks involved. This legitimacy must always be conditioned by procedural safeguards, regular review, and multidisciplinary involvement in order to minimize arbitrariness and preserve the primacy of care over control [115].

A pivotal criterion in this evaluation is the patient’s mental capacity. When decisional capacity is preserved, the use of coercion is ethically unjustifiable. When decisional capacity is preserved, the use of coercion is ethically unjustifiable. However, when capacity is severely impaired due to acute psychopathology, temporary and proportionate restrictions may be deemed necessary in order to protect both the individual and others. Capacity assessment is therefore a safeguard for autonomy and dignity, preventing coercion from becoming a substitute for clinical or social inadequacy [116].

This ethical stance is not a recent development; it constitutes one of the fundamental legacies of the Italian Basaglia Law, which foreshadowed by decades the present international debates on autonomy, vulnerability, and the relational nature of care. By grounding psychiatric practice on the recognition of dignity rather than on social dangerousness alone, the Basaglia framework established a normative and ethical pillar that remains crucial today. The proposal under consideration seeks to operationalize this foundation by integrating capacity-based evaluation with intermediate measures and collegial governance. Consequently, even within coercive contexts, decisions are not left to individual discretion but are embedded in a transparent and shared responsibility. This approach is predicated on the principle of justice in the distribution of risks and burdens, while preserving the ethical imperative that, even under constraint, care must remain anchored to respect for the person.

Significantly, this model is consistent with the goals set out in the World Health Organization’s Comprehensive Mental Health Action Plan 2013–2030 [117], which calls for the development of inclusive, community-based, and rights-oriented mental health systems. The proposed governance framework provides a concrete operational response to these international objectives, promoting equity, patient dignity, and institutional responsibility for delivering safe and continuous care. Pilot implementation and systematic evaluation of this model could represent a decisive step forward in modernizing and structurally strengthening Italy’s public psychiatric system, as well as a significant contribution to the international pursuit of ethically sound, equitable, and sustainable mental health governance.

### 6.2. Disability in a Broad Sense and Opportunity for Convergence

Italy’s Disability Reform (Legislative Decree 62/2024), enacted in June 2024, adopts the UN Convention’s broad conception of disability as the result of the interaction between long-term health conditions and environmental barriers, moving well beyond a purely medical or impairment-based view [118].

By establishing a single, multidimensional assessment that culminates in a personalized “life plan” the decree creates an institutional ecosystem that aligns with our three- pillar model. The Multidimensional Assessment Teams envisioned for the general disabled population could serve as the gatekeeping mechanism for high-complexity psychiatric cases. At the same time, our Multidisciplinary Board would provide the longitudinal risk governance that the decree currently lacks.

This convergence presents a unique policy window: aligning mental health reform with the broader disability overhaul reduces duplication, streamlines administrative pathways, and strengthens the equity of access.

### 6.3. Limitations of the Proposal

While the proposed framework is theoretically coherent and structurally innovative, it presents five main limitations. First, its implementation would require financial and professional resources that are currently unavailable in many areas of the Italian healthcare system, which has long suffered from underfunding and structural deficiencies. This challenge is compounded by an organizational culture often geared toward reactive crisis management rather than systemic, forward-looking planning—an orientation that may hinder the adoption of a structured, multilayered, and continuity-based model.

Second, the involvement of the Guardianship Judge in the Multidisciplinary Board, though consistent with the principle of judicial oversight, risks remaining ineffective without procedural reforms to enhance its operational role. In current practice, this function is even more limited than in the proposed model, hindered by the substantial workload already cited by judicial authorities as a barrier to conducting in-person hearings for the validation of compulsory admissions (TSO).

As things presently stand, the judge’s role is largely symbolic, exerting minimal influence on clinical and organizational decisions. A third limitation concerns the absence of empirical data on patients’ perspectives. Although the model draws inspiration from the principle of relational autonomy, the lack of direct patient input, participatory consultation, or qualitative assessments weakens its ethical and social legitimacy. This gap reflects a broader structural absence in the Italian context but could be addressed through systematic monitoring mechanisms, including tools designed to assess acceptability, perceived effectiveness, and the quality of therapeutic relationships.

Furthermore, Forensic Psychiatry Units—despite being conceived as community-based, outpatient, and healthcare-oriented structures—may be perceived as a veiled reintroduction of a custodial approach reminiscent of the former forensic psychiatric hospitals (OPG). Without clear legal definitions and transparent communication, such perceptions may generate institutional and cultural resistance, particularly in light of Italy’s collective memory of the custodial era.

Lastly, the implementation of Community Treatment Orders, while modeled on international practices, raises ethical and legal concerns. In the absence of strict eligibility criteria, systematic outcome evaluation, and use limited to high-complexity cases, this measure may inadvertently legitimize coercive interventions that lack robust evidence of effectiveness, thereby compromising the principles of proportionality and protection of fundamental rights.

### 6.4. Implications for Practice

We propose, in selected regions, a pilot initiative of outpatient treatment under judicial oversight with clear safeguards: judicial authorization, time limits, rights information, periodic reviews, an explicit sunset clause, and predefined recall criteria. At each Local Health Authority, a multidisciplinary committee would deliberate on the most complex cases and ensure shared accountability between clinicians and management, with the involvement of representatives of the judiciary. Care transition pathways from enhanced-security units to acute wards, and then to community services, would follow time-bound objectives, with proportionate recall or readmission procedures to reduce revolving-door admissions and prevent inappropriate referrals to forensic settings. The organizational arrangement is designed to lighten clinicians’ administrative burden through collegial structures, standardized discharge plan templates, a legal coordination function, and a dedicated data lead responsible for indicators and audit.

## 7. Conclusions

High-complexity psychiatric cases, in which clinical severity intersects with legal and social vulnerability, often exceed the decision-making capacity of individual practitioners and may jeopardize both patient safety and quality of care. A three-pillar governance model, combining intermediate legal instruments, dedicated Forensic Psychiatry Units, and a second-tier Multidisciplinary Board, offers a concrete opportunity to reconcile individual liberty, social protection, and medico-legal accountability. Its pilot implementation and systematic evaluation could mark a decisive step toward modernizing and structurally reinforcing Italy’s public mental health system.

## Figures and Tables

**Table 1:** Highly complex psychiatric patients.

For the purpose of this analysis, highly complex psychiatric patients are defined as individuals who meet one or more of the following criteria:
Clinical signs of imminent or repeated violence towards others.
Current or recent suicidal ideation or attempt with intent or planning.
Severe self-neglect jeopardizing physical safety or health.
Marked illness instability with poor insight and repeated treatment non-adherence.
Justice system involvement (e.g., court-supervised psychiatric measures or recent discharge from secure forensic facilities).
Active substance use disorder.
This definition draws from existing clinical tools for structured risk assessment and is tailored to reflect the population gaps within the Italian governance framework.

**Table 2:** Involuntary admission and intermediate/step-down pathways.

Country	Acute Phase/Max Legal Duration	Intermediate/Step-Down	Community Compulsory Treatment
**Italy**	TSO: 7-day initial involuntary hospitalization; renewable in 7-day blocks; judge validation ≤ 48 h	CMHC follow-up, day hospital, residential facilities (where available)	**No**
**England and Wales**	Section 2: up to 28 days; Section 3: up to 6 months, renewable	PICU where required; Section 117 aftercare; CMHT	Yes: CTO (Section 17A), 72 h recall
**France**	SPDT/SPPI/SPDRE validated by day 12; renewable every 6 months	*Programmes de soins* (outpatient)	**Yes:** Involuntary outpatient via *Programmes de soins* (psychiatrist-ordered)
**Germany**	Judge hearing/confirmation ≤ 24 h; civil orders typically 6 weeks → 6 months → 1 year; renewable	Periodic judicial review; arrangements vary by Land	**No**
**Spain**	Urgent: judge notified ≤ 24 h, decision ≤ 72 h; no fixed maximum; periodic review (≥6 months; some regions 2 months)	Possible outpatient conversion; ad hoc IOT in some regions	**Mixed:** IOT ad hoc (no nationwide statute)
**Canada**	Emergency hold 24–72 h; admission certificate 14–30 days; renewals 30 × 2 → 90 → 180 days	Step-down with community supports; Review Boards in many provinces	**Yes:** CTO (6 months, renewable; police recall; nine provinces)
**USA**	Emergency hold 24–72 h; short-term ≈ 14 days; long-term commonly 90+ days (state-dependent)	Varies by state; conditional release/step-down programs	**Yes:** AOT (6–12 months; brief rehospitalization on non-compliance)

Legend: AOT (Assisted Outpatient Treatment); CMHC (Community Mental Health Center); CMHT (Community Mental Health Team); CTO (Community Treatment Order); TSO (Compulsory Health Treatment Order); IOT (involuntary outpatient treatment); PICU (Psychiatric Intensive Care Unit); SPDT (*Soins psychiatriques à la demande d’un tiers*); SPPI (*Soins psychiatriques en péril imminent*); SPDRE (*Soins psychiatriques sur décision du représentant de l’État*).

**Table 3:** Forensic psychiatric measures.

Country	Forensic Legal Measure	Oversight, Review, and Duration/Discharge
**Italy**	REMS placement after insanity acquittal (security measure); length ≤ statutory maximum for the index offense.	Surveillance judiciary (magistrate/tribunal); periodic review of dangerousness as required by law; duration constrained by the penal maximum; discharge or step-down decided by the surveillance judiciary.
**England and Wales**	Hospital Order (“s.37”) ± Restriction Order (“s.41”); also pre-sentence orders (“ss.35/38”), prison transfer orders (“ss.47/48”) ± Restriction Direction (“s.49”), and the hybrid order (“s.45A”).	MoJ oversight for restricted patients; First-tier Tribunal (Mental Health) appeals; conditional discharge with liability to recall; Hospital Orders (with or without restriction) are indeterminate, subject to periodic risk review; transfer delays from prison may occur due to bed availability.
**France**	Three-level prison mental health system: (I) USMP (in-prison ambulatory psychiatric care). (II) SMPR (in-prison day-time psychiatric hospitalization). (III) UHSA: full-time hospitalization within the prison system (admission may be voluntary or without consent). UMD: civil high-security units for high-risk/difficult-to-treat patients. Post-custodial measures: *rétention de sûreté*/*surveillance de sûreté*.	JLD validation and annual review where care is without consent; specialized criminal justice oversight for penal security measures; custodial/supervision measures are renewable per statute with possible step-down to community supervision.
**Germany**	§63 StGB (hospitalization of offenders lacking criminal responsibility); §64 StGB (forensic addiction treatment alongside a custodial sentence); §66 StGB (preventive detention).	Criminal courts; review intervals: §63 annually, §64 every 6 months, §66 every 2 years. Durations: §63 indeterminate (review-based); §64 up to 2 years plus two-thirds of the parallel prison sentence, executed before the custodial term with transfer back to prison if treatment fails; §66 indeterminate preventive detention with periodic review.
**Spain**	*Internamiento* in psychiatric/detox/educational facilities for non-imputable defendants (Criminal Code 101–103).	Annual judicial reconfirmation; may exceed custodial maximum by up to 5 years per statute; convertible to outpatient treatment as risk decreases; time credited to sentence.
**Canada**	NCRMD → Provincial Review Board: detention, conditional discharge, or absolute discharge.	Provincial Review Boards; annual risk-based reviews; least-restrictive principle; discharge determined by current risk.
**USA**	NGRI commitment to state forensic hospital; GBMI in some jurisdictions.	Court/board hearings with probable-cause/full evidentiary reviews and periodic re-evaluation; NGRI detention until no longer dangerous; GBMI = penal custody with treatment.

Legend: GBMI (Guilty But Mentally Ill); JLD (*Juge des libertés et de la détention*); MoJ (Ministry of Justice); NCRMD (Not Criminally Responsible on account of Mental Disorder); NGRI (Not Guilty by Reason of Insanity); REMS (Residences for the Execution of Security Measures); SMPR (*Service Médico-Psychologique Régional*); StGB (German Criminal Code); UMD (*Unités pour Malades Difficiles*); UHSA (*Unités Hospitalières Spécialement Aménagées*); USMP (*Unités Sanitaires en Milieu Pénitentiaire*).

## Data Availability

The original contributions presented in this study are included in the article and its Supplementary Materials. Further inquiries can be directed to the corresponding author.

## Supplementary Materials

The following supporting information can be downloaded at: https://www.mdpi.com/article/10.3390/healthcare13182363/s1. Supplementary Methods File S1: Detailed methodology; Table S1: Detailed data on annual national spending on mental health in Italy (2015–2023).
